# Roles of increased glycaemic variability, GLP-1 and glucagon in hypoglycaemia after Roux-en-Y gastric bypass

**DOI:** 10.1530/EJE-17-0446

**Published:** 2017-08-30

**Authors:** George Tharakan, Preeshila Behary, Nicolai J Wewer Albrechtsen, Harvinder Chahal, Julia Kenkre, Alexander D Miras, Ahmed R Ahmed, Jens J Holst, Stephen R Bloom, Tricia Tan

**Affiliations:** 1 Division of DiabetesEndocrinology and Metabolism, Imperial College London, London, UK; 2 NNF Center for Basic Metabolic Research and Department of Biomedical SciencesFaculty of Health and Medical Sciences, University of Copenhagen, Copenhagen, Denmark; 3 Department of SurgeryImperial College Healthcare NHS Trust, London, UK

## Abstract

**Objective:**

Roux-en-Y gastric bypass (RYGB) surgery is currently the most effective treatment for diabetes and obesity. An increasingly recognized and highly disabling complication of RYGB is postprandial hypoglycaemia (PPH). The pathophysiology of PPH remains unclear with multiple mechanisms suggested including nesidioblastosis, altered insulin clearance and increased glucagon-like peptide-1 (GLP-1) secretion. Whilst many PPH patients respond to dietary modification, some have severely disabling symptoms. Multiple treatments are proposed, including dietary modification, GLP-1 antagonism, GLP-1 analogues and even surgical reversal, with none showing a more decided advantage over the others. A greater understanding of the pathophysiology of PPH could guide the development of new therapeutic strategies.

**Methods:**

We studied a cohort of PPH patients at the Imperial Weight Center. We performed continuous glucose monitoring to characterize their altered glycaemic variability. We also performed a mixed meal test (MMT) and measured gut hormone concentrations.

**Results:**

We found increased glycaemic variability in our cohort of PPH patients, specifically a higher mean amplitude glucose excursion (MAGE) score of 4.9. We observed significantly greater and earlier increases in insulin, GLP-1 and glucagon in patients who had hypoglycaemia in response to an MMT (MMT Hypo) relative to those that did not (MMT Non-Hypo). No significant differences in oxyntomodulin, GIP or peptide YY secretion were seen between these two groups.

**Conclusion:**

An early peak in GLP-1 and glucagon may together trigger an exaggerated insulinotropic response to eating and consequent hypoglycaemia in patients with PPH.

## Introduction

The Roux-en-Y gastric bypass (RYGB) operation results in a rapid improvement in any pre-existing type 2 diabetes mellitus (T2DM) that occurs shortly after the operation prior to any significant weight loss (1, 2). Whilst the cause for this remains unclear, it is apparent that there is a dramatic alteration in glucose tolerance. Beyond a reduction in fasting and postprandial plasma glucose levels, there is also a change in the glucose tolerance curve. Instead of a steady increase in glucose that plateaus before returning to baseline, there is a sharp rise with a peak glucose at 30 min with a subsequent rapid drop (3).

Glycaemic variability (GV) is broadly defined as the fluctuations between hyper and hypoglycaemia that typically occur in diabetes (4). There is also an increase in GV post-RYGB, relative to obese subjects who have not had RYGB surgery (5, 6, 7, 8, 9). Increased GV is not exclusive to RYGB patients with diabetes but is also observed in patients who are considered euglycaemic both before and after surgery (3, 10). It is now recognised that a proportion of patients after surgery develop disabling symptoms of postprandial hypoglycaemia (PPH) that are associated with this increased GV.

The reported prevalence of PPH varies from 0.1% (severe cases) to 13.3% (mild hypoglycaemia) but is likely under-reported due to a combination of vague symptoms, a lack of awareness of this condition and lack of agreement on diagnostic standards (10, 11). PPH after RYGB was first described by Service *et al.* in 2005 in 6 patients (12). However, cases of PPH occurring after partial gastrectomies for gastric ulcer disease have been reported since the 1930s (13). Understanding the pathophysiology is important as the increasing prevalence of patients who have undergone surgery means that PPH will become more and more common with time (14). Additionally, whilst it is established that severe hypoglycaemia can result in seizure or coma and even death, it is increasingly recognised that even mild hypoglycaemia can predispose to cardiac arrhythmias (15). Therefore, it is critical that PPH is recognised and treated.

The causes of PPH remain unclear. It has been long established that the hypoglycaemia is hyperinsulinaemic in nature but the underlying mechanism remains unknown. Previous work has focused on incretins (insulinotropic hormones that are secreted in response to an oral glucose load) and the exaggerated secretion of the incretin glucagon-like peptide-1 (GLP-1) (16). Some studies have shown elevated GLP-1 levels in patients with PPH relative to patients without PPH whilst others have failed to confirm this (16, 17, 18). Furthermore, both GLP-1 receptor antagonists and agonists have been shown to improve PPH, which is difficult to reconcile (19, 20).

To address these areas of uncertainty regarding the pathophysiology of PPH, we report our experience within the Imperial Weight Centre (IWC). We describe the dynamics of gut hormone secretion, notably peptide YY (PYY), GLP-1, glucagon and oxyntomodulin (OXM) and glycaemic variability in a cohort of patients referred to the IWC with PPH who underwent a mixed meal test (MMT) and CGM (continuous glucose monitoring).

## Subjects and methods

### Study design and participants

Patients with symptomatic PPH were identified from cases referred to the IWC. All patients were shown to experience episodes fulfilling Whipple’s triad for hypoglycaemia. Patients with fasting hypoglycaemia <3.0 mmol/L were excluded. Three of the RYGB patients had had surgery at another bariatric centre but were referred to the IWC for management of their symptomatic PPH. All subjects were assessed by an experienced physician to exclude alternative causes such as epilepsy or postural orthostatic tachycardia syndrome prior to further investigation.

One of the key issues with PPH is that patients do not consistently report symptoms with one type of meal or other provocation, and even when patients are consistently taking the same meal on different occasions, hypoglycaemia may not always occur. In the absence of a gold-standard diagnostic test, we took the approach that we would administer a standardised provocation test in the form of a liquid mixed meal test (MMT) with a fixed carbohydrate, fat and protein content. To determine if changes in gut hormone secretion in response to this trigger are associated with biochemical hypoglycaemia, these changes were compared between subjects that had biochemical hypoglycaemia during a MMT (MMT Hypo) and those that did not experience hypoglycaemia (MMT Non-Hypo).

Further control groups (obese subjects who have not undergone RYGB surgery and RYGB patients who were asymptomatic from PPH) were obtained from an on-going randomised controlled study currently being performed at Imperial College London (REC reference no: 13/LO/1510).

### Continuous glucose monitoring (CGM)

Subjects underwent a CGM study for 5 days, under free living conditions. Initially a Meditronic iPro2 system (Meditronic, Northridge, USA) was used, and in later studies, this was changed to Abbott FreeStyle Navigator II (Abbott). For both types of CGM, subjects were required to calibrate their machines with capillary blood glucose testing. For the Meditronic iPro2 system, four calibrations a day were performed whilst the Abbott FreeStyle Navigator II required calibration after 1, 2, 10, 24 and 72 h following sensor insertion. Data were analysed using the easyGV calculator for measures of GV (http://www.phc.ox.ac.uk/research/technology-outputs/easygv; accessed: 6 January 2016). Measures of GV analysed included: standard deviation (s.d.), lability index (LI), mean of daily differences (MODD), mean amplitude glucose excursion (MAGE), glycaemic risk assessment in diabetes equation (GRADE), continuous overlapping net glycaemic action (CONGA), high blood glucose index (HBGI), low blood glucose index (LBGI), average daily risk ratio (ADRR) and mean absolute glucose (MAG).

### Mixed meal test (MMT)

Subjects arrived at the investigation unit at 08:30 h having fasted overnight. A peripheral venous cannula was inserted for blood sampling. At 09:00 h, subjects consumed an Ensure Plus food supplement (13.8 g of protein, 10.8 g of fat, 44.4 g of carbohydrates, 330 kcal, 220 mL, Abbott) within 10 min. Blood sampling for glucose, insulin, C-peptide and gut hormones were taken at thirty-minute intervals for four hours. In addition, a capillary blood glucose (CBG) sample was taken if subjects complained of hypoglycaemic symptoms. The study was stopped if subjects developed neurological symptoms of hypoglycaemia and had a CBG of ≤3.0 mmol/L. Biochemical hypoglycaemia was defined as a venous plasma glucose ≤3.0 mmol/L in keeping with the consensus guidelines on hypoglycaemia (21). The insulinogenic index was calculated as the ratio of incremental insulin concentration to glucose concentration at 30 min (22).

Glucose and insulin were measured by the Department of Chemical Pathology, Imperial College Healthcare NHS Trust using an Abbott Architect integrated system analyser with coefficients of variation (CV) of <5% and <7% respectively. Plasma samples for gut hormones were collected in lithium heparin tubes containing aprotinin (1000 kallikirein inhibitor units). Plasma total PYY and GIP were measured using the Milliplex magnetic bead-based multi-analyte, metabolic panel, 4-plex immunoassay (Millipore). The CV for each analyte was <10% and <15% with reference to intra- and inter-assay precision. The lowest level of quantification was 3.4 and 0.3 pmol/L for total PYY and GIP respectively. OXM was measured by a radioimmunoassay validated by mass spectroscopy as described in a previous study (23). The plasma total GLP-1 and glucagon were measured using ELISAs from Mercodia (Uppsala, Sweden), with lowest levels of detection of 1.0 and 1.5 pmol/L respectively. The cross-reactivity of the glucagon assay for OXM and GLP-1 was <4.4% and <0.3%. Quality controls co-delivered with the kits and run in parallel with the plasma samples were all within prespecified limits.

### Statistical analysis

Statistical analysis was performed using Prism 6.0 (GraphPad Software). Two-way repeated measures ANOVA with Bonferroni *post hoc* test was used to compare differences in glucose, insulin, total GLP-1, GIP, PYY and glucagon at different time points. One-way ANOVA with Tukey *post hoc* test was used to compare the differences in AUCs when more than two groups were being compared. Unpaired Student *t*-tests were used to compare differences between groups when only two groups were being compared. Normality of data was assessed by a D’Agostino–Pearson test. Comparisons of measures of glycaemic variability that did not have a Gaussian distribution was performed using a Wilcoxon test. Results are presented as mean ± s.e.m. and statistical significance defined as *P* < 0.05.

## Results

The demographics of the recruited subjects are shown in Table 1. Eighteen subjects had symptomatic PPH (Table 2). Eight out of the 18 patients had T2DM prior to surgery, but all were in remission (as defined by the American Diabetes Association) after surgery (24). All patients had had RYGB at least 1 year prior to study. Two control groups were also analysed; a group of obese patients who had not had RYGB surgery (obese no-RYGB, *N* = 9) and a group consisting of RYGB patients who had not had symptoms of PPH (asymptomatic RYGB, *N* = 10).

**Table 1:** Demographics of all subjects in study. Data is shown as mean ± s.e.m.

	**Symptomatic PPH**	**Obese-No RYGB controls**	**Asymptomatic RYGB controls**
Number of subjects	18	9	10
Gender	10F, 8M	7F, 2M	9F, 1M
Age (years)	47.5 ± 2.4	43.6 ± 3.9	46.7 ± 4.2
HbA1c (mmol/mol)	38.4 ± 1.2	39.8 ± 1.7	39.2 ± 1.4
Presurgical weight (kg)	130.1 ± 4.6		131.4 ± 3.5
Percentage weight loss at 1 year (%)	32.2 ± 1.6		31.1 ± 2.3

F, female; M, male.

**Table 2:** Demographics of subjects with PPH.

**Patient No.**	**Year of surgery**	**Type of surgery**	**Age** (years)	**Diabetes pre surgery**	**Presurgical weight** (kg)	**Percentage weight loss at 1 year** (%)	**Onset of symptoms from surgery** (years)
1	2010	RYGB	50	Yes	117	32	2
2	2010	RYGB	60	No	118	28	1
3	2011	RYGB	52	Yes	127	35	2
4	2012	RYGB	46	Yes	118	29	1
5	2011	RYGB	43	Yes	153	43	2
6	2013	RYGB	33	No	138	33	1
7	2007	RYGB	51	No	138	33	6
8	2010	RYGB	52	No	130	37	1
9	2007	RYGB	44	Yes	126	41	3
10	2007	RYGB	26	No	151	27	3
11	2010	RYGB	44	Yes	131	33	2
12	2010	RYGB	51	Yes	98	38	1
13	2012	RYGB	46	No	167	31	1
14	2010	RYGB	71	Yes	121	36	3
15	2013	RYGB	33	No	168	25	1
16	2012	RYGB*	55	No	121	12	2
17	2014	RYGB	48	No	104	33	1
18	2012	RYGB	50	No	115	34	3

* conversion from sleeve RYGB, Roux-en-Y Gastric Bypass.

Eleven subjects (patient numbers 8–18 from Table 2) with symptoms of PPH post-RYGB wore CGMs for five days. Their data was compared to a reference range for non-diabetic patients who have not had RYGB (4). The PPH patients had MAGE, ADDR and MAG values that were higher than the reference range (Table 3). Of these 11 subjects, five developed hypoglycaemia during the MMT. When those patients that developed hypoglycaemia during the MMT were compared to those that did not develop hypoglycaemia during the MMT, there was no significant difference in measures of GV or proportion of time in hypoglycaemia between the two groups (data not shown). A further analysis was performed comparing glycaemic variability during the hours of 06:00–22:00 (representing the awake period) to 22:00–06:00 (representing the sleep period). Glycaemic variability was increased in the day time relative to night time for all measures except for GRADE (Table 3).

**Table 3:** Measurement of parameters of glycaemic variability in PPH patients.

		**Symptomatic PPH** (*N* = 11)		
**GV measure**	**Reference range**	Awake (06:00–22:00)	Asleep (22:00–06:00)	Total
s.d.	0.0–3.0	1.8 ± 0.7**	1.2 ± 0.1	1.8 ± 0.2
CONGA	3.6–5.5	4.8 ± 0.1***	4.2 ± 0.1	4.6 ± 0.2
LI	0.0–4.7	5.1 ± 0.6**	3.1 ± 0.7	4.7 ± 0.8
J-INDEX	4.7–23.6	18.8 ± 1.2***	13.8 ± 1.1	18.4 ± 1.9
LBGI	0.0–6.9	3.8 ± 0.7**	5.4 ± 0.8	4.9 ± 1.1
HBGI	0.0–7.7	3.5 ± 0.5**	1.8 ± 0.4	3.4 ± 0.7
GRADE	0.0–4.6	2.2 ± 0.9	1.8 ± 0.4	1.2 ± 0.2
MODD	0.0–3.5	N/A	N/A	1.5 ± 0.1
MAGE	0.0–2.8	5.0 ± 0.4***	2.6 ± 0.3	4.9 ± 0.4
ADDR	0.0–8.7	N/A	N/A	12.0 ± 2.9
*M* Value	0.0–12.5	6.7 ± 1.5*	10.1 ± 2.6	9.4 ± 2.7
MAG	0.5–2.2	2.3 ± 0.1*	1.8 ± 0.1	2.3 ± 0.2
% time in hypoglycemia <3.0		2.4 ± 1.4	1.7 ± 1.1	2.0 ± 0.9
% time in range, 3.0–7.0		76.0 ± 4.0	86.8 ± 2.7	81.4 ± 2.6
% time in hyperglycemia >7.0		21.6 ± 3.7	11.6 ± 2.8	16.6 ± 2.5

* *P* value <0.01, **P value <0.001 and ***P value <0.0001.

ADRR, average daily risk ratio; CONGA, continuous overlapping net glycaemic action; GRADE, glycaemic risk assessment in diabetes equation; HBGI, high blood glucose index; LBGI, low blood glucose index; LI, lability index; MAG, mean absolute glucose; MAGE, Mean Amplitude Glucose Excursion; MODD, mean of daily differences; s.d., standard deviation.

**Figure 1:**
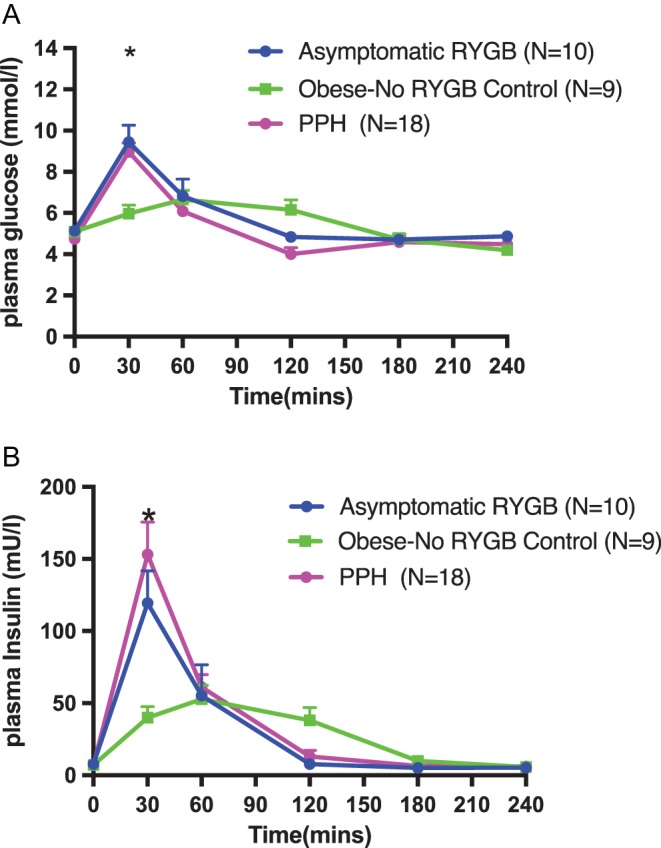
Changes in plasma glucose (A) and serum insulin (B) following a MMT in patients with symptomatic PPH, a non-surgical obese group and an asymptomatic RYGB group. Mixed meal was given at *T* = 0. Numbers of subjects in each group shown in brackets. Data is shown as mean ± s.e.m. Statistically significant differences are shown as *PPH vs Obese-No RYGB *P* < 0.001, asymptomatic-RYGB vs Obese-No RYGB *P* < 0.001. **PPH vs Obese-No RYGB *P* < 0.001.

Eighteen symptomatic PPH subjects and 19 subjects from the two control groups underwent a MMT (Fig. 1, Supplementary Tables 1 and 2, see section on supplementary data given at the end of this article). There was no significant difference in the fasting glucose concentration between the three groups. Thirty minutes following the consumption of the mixed meal, there was a significant difference in plasma glucose concentration between both surgical cohorts relative to Obese-No RYGB group (glucose_30_: 9.0 ± 0.4 (symptomatic PPH group), 9.4 ± 0.8 (asymptomatic RYGB), 6.0 ± 0.4 mmol/L (Obese-No RYGB) – *P* < 0.0001 both for comparison of symptomatic PPH vs Obese-No RYGB and for comparison of asymptomatic RYGB vs Obese-No RYGB). Plasma glucose levels fell to baseline by 120 min and there appeared to be no significant difference in mean glucose levels at this timepoint between the PPH and asymptomatic RYGB groups (Glucose_120_: 4.8 ± 0.2 (asymptomatic RYGB) vs 4.0 ± 0.3 mmol/L (symptomatic PPH group), *P* = NS for comparison).

There was no significant difference in the fasting concentration of insulin between all three groups. Thirty minutes following the consumption of the mixed meal, there was a significant difference in serum insulin concentration between both surgical cohorts relative to Obese-No RYGB group (Insulin_30_: 132.0 ± 24.8 (symptomatic PPH group), 119.4 ± 24.9 (asymptomatic RYGB), 39.9 ± 7.4 U/L (Obese-No RYGB – *P* < 0.0001 both for comparison of symptomatic PPH vs Obese-No RYGB and for comparison of asymptomatic RYGB vs Obese-No RYGB). In both RYGB groups, the serum insulin returned to baseline at 120 min whilst the Obese-No RYGB group’s serum insulin was more persistently elevated at 120 min (insulin_120_:7.8 ± 1.1 (asymptomatic RYGB) vs 13.0 ± 4.2 (symptomatic PPH group) vs 38.1 ± 8.9 U/L (Obese-No RYGB) – *P* = NS for comparisons between groups).

Seven of the 18 PPH patients had plasma glucose concentrations below 3.0 mmol/L between 60 and 120 min following an MMT. To further investigate the gut hormone dynamics that trigger PPH, the data were categorised into those that had biochemical hypoglycaemia of <3.0 mmol/L during the course of the MMT (MMT Hypo) and those that did not (MMT Non-Hypo). Whilst the seven MMT Hypo patients were symptomatic, none of them developed severe neurological symptoms that required treatment.

**Figure 2:**
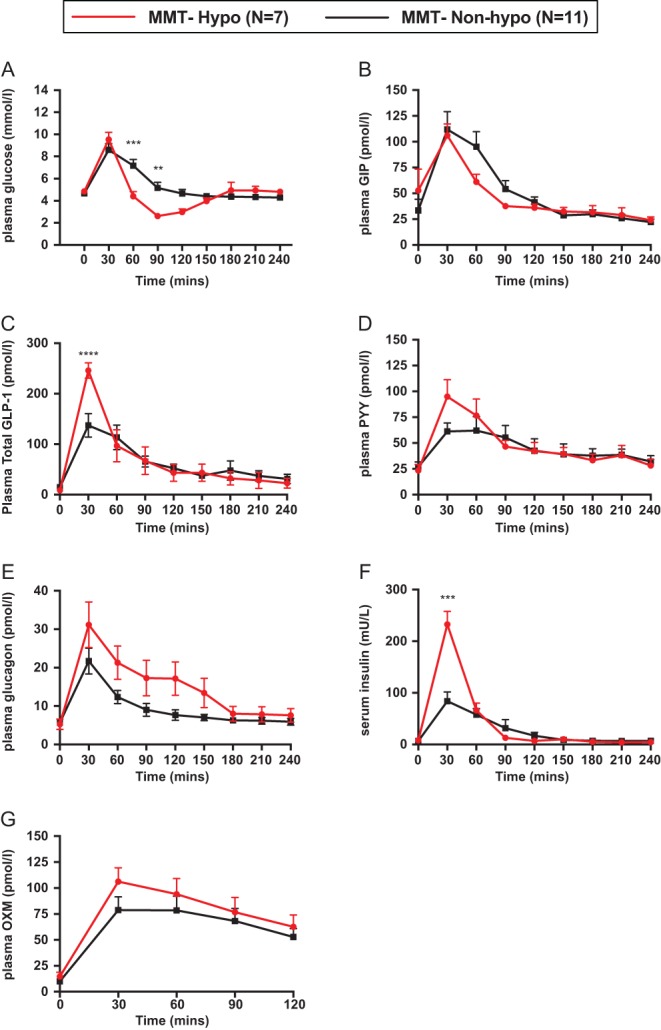
Changes in glucose (A) and insulin (B) serum GLP-1 (C), GIP (D), glucagon (E), peptide YY (F), and OXM (G) following a MMT in patients with symptomatic PPH. Patients classified as either MMT Hypo (*n* = 7) or MMT Non-Hypo (*n* = 11), depending if their nadir glucose was <3 mmol/L. Data is shown as mean ± s.e.m. Statistically significant differences are shown as **P < 0.01, ***P < 0.0001.

Glucose and insulin responses to the MMT are shown in Fig. 2 and Supplementary data. The peak glucose was not different between the MMT Hypo and Non-Hypo group (9.5 ± 0.7 (MMT Hypo group) vs 9.2 ± 0.4 mmol/L (MMT Non-Hypo group)). As expected, the MMT Hypo group had a significantly lower nadir glucose relative to the MMT Non-Hypo group (nadir glucose 2.4 ± 0.2 (MMT Hypo group) vs 3.9 ± 0.1 mmol/L (MMT Non-Hypo group)). With reference to the glycaemic profile, there was no other significant difference between the two groups.

There was no significant difference in the baseline insulin concentration between the two groups. However, after consuming the mixed meal, the MMT Hypo group had a significantly higher mean peak serum insulin concentration at 30 min (insulin_30_ 232.7 ± 25.0 (MMT Hypo group) vs 102.2 ± 22.7 U/L (MMT Non-Hypo group) *P* < 0.05). There was no significant difference in the AUC insulin but the MMT Hypo group had a higher insulin response relative to their plasma glucose as evidenced by a significant difference in the insulinogenic index and the ratio of AUC insulin: glucose (Supplementary Table 2).

Baseline fasting glucagon levels were not significantly different between the MMT Hypo group and the MMT Non-Hypo group (5.3 ± 1.3 (MMT Hypo group) vs 5.6 ± 0.7 pmol/L (MMT Non-Hypo group) – Fig. 2E). Following ingestion of the mixed meal, the glucagon concentration increased in both the MMT Hypo group at 30 min (glucagon_30_ 31.1 ± 6.0 (MMT Hypo group) vs 21.7 ± 3.4 pmol/L (MMT Non-Hypo group), *P* = 0.07) and at 60 min (glucagon_60_ 21.3 ± 4.3 (MMT Hypo group) vs 12.4 ± 1.7 pmol/L (MMT Non-Hypo group), *P* = NS). Although there was a trend towards elevated glucagon levels at 30 min in the MMT Hypo group compared to the MMT Non-Hypo group, this difference was not statistically significant. However, the mean AUC glucagon for the MMT Hypo group was significantly higher (3677 ± 689.5 pmol/L.min) compared to the MMT Non-Hypo group (2279 ± 230.3 pmol/L.min, unpaired *t*-test, *P* = 0.0368).

Baseline fasting total GLP-1 levels were also not significantly different between the MMT Hypo and the MMT Non-Hypo groups (8.7 ± 2.5 (MMT Hypo group) vs 14.9 ± 3.7 (MMT Non-Hypo group) – Fig. 2C). Following ingestion of the mixed meal, the GLP-1 concentration increased in both the MMT Hypo and MMT Non-Hypo groups at 30 min (GLP-1_30_ 246.0 ± 15.3 (MMT Hypo group) vs 137.3 ± 23.4 pmol/L (MMT Non-Hypo group), *P* < 0.0001), but significantly more in the MMT Hypo group.

There was no significant difference in concentrations of total PYY, GIP and OXM at any time point during the MMT between both groups, nor in the AUC (Fig. 2B, D and G).

## Discussion

This study has demonstrated the altered physiology of glucose handling that occurs after RYGB surgery through two different clinical tests, continuous glucose monitoring and mixed meal testing. For our particular study, we have chosen a pragmatic definition of PPH which is based on the clear demonstration of biochemical hypoglycaemia in association with Whipple’s triad. Previous studies have compared bariatric surgery patients known to have symptomatic PPH with a group of asymptomatic patients. Salehi *et al.* have previously demonstrated a significantly lower glucose nadir in those that have a history of PPH (25), but this was not replicated in a subsequent study by the same authors (18) or by others such as Goldfine *et al.* (16) and Laurenius *et al.* (17). This disparity between studies is explained by the failure of the provocation test used to induce hypoglycaemia in all the PPH patients whilst some within the asymptomatic group developed biochemical hypoglycaemia without symptoms (18, 25). This illustrates two important observations. First, in the RYGB cohort, there is a high prevalence of asymptomatic hypoglycaemia. This may be due to hypoglycaemic unawareness and attenuated counter-regulatory responses in patients post RYGB (26). Secondly, in patients with PPH, the reproduction of this condition with dynamic testing appears variable. In our cohort, only 7 of 18 were shown to have biochemical hypoglycaemia after a mixed meal provocation. For non-bariatric patients who are suspected to have PPH, the MMT is recommended as the gold standard test (21). On the other hand, there is no gold standard test to diagnose PPH in bariatric surgery patients and within the literature many tests have been used such as prolonged oral glucose tolerance test, MMT and CGM (17, 25).

### Altered glycaemic variability post RYGB

Subjects with symptomatic PPH were shown to have increased GV as measured by CGM, although there was no difference in measures of GV between the subgroup that experienced a hypo in response to the MMT vs those that did not. Specifically, MAGE, ADDR and MAG were increased relative to normal limits defined by Hill *et al.* in 70 non-RYGB operated euglycaemic subjects (21). Similar findings were made in a separate study by Hanaire *et al.* who demonstrated an increase in MAGE in 10 PPH patients of 4.8 ± 3.3 (8).

MAGE and MAG assess glucose excursions from the mean glucose level with the former designed specifically for postprandial changes. ADDR assesses the prevalence of glucose values outside the normal range and hence is a good predictor for both hyper- and hypoglycaemia (27). The changes in these parameters of GV highlight the altered glucose tolerance observed after RYGB surgery, which is seen in the data from the MMT. GV, in our cohort of patients, was significantly higher as assessed by multiple parameters during 06:00–22:00 (awake period) compared to 22:00–06:00 (asleep), possibly due to differences in the diurnal rhythms of metabolism and hormone secretion, physical activity and food intake. A limitation of the present analysis is that no specific data were collected to enable an analysis of the relationship of exercise and food intake to glycaemia, and future studies should examine any impacts of these factors on the development of PPH.

### Altered glucose tolerance in patients post RYGB measured by a MMT

This study has demonstrated that patients post-RYGB have an altered glucose tolerance relative to a control group of obese euglycaemic individuals, independently of whether they have PPH. The altered glucose tolerance is characterised by significantly earlier and higher glucose peak concentration following consumption of a mixed meal. These data are consistent with previous reports (3).

The higher peak concentration of glucose in the RYGB patients is in keeping with accelerated nutrient entry and absorption after bypass (28). Although all the studied RYGB patients had normal glycated haemoglobin, they had an increase in GV which has been shown to be an independent risk factor for an increase in microvascular complications (29). Miras *et al.* in a prospective case series demonstrated an improvement in nephropathy at one year post surgery, but this was not shown for retinopathy or neuropathy (7). Furthermore, a recent meta-analysis investigating the effects of bariatric surgery on retinopathy revealed that whilst 19.2 ± 12.9% of patients had improvements, 23.5±18.7% had a deterioration in retinopathy (9). More reassuringly, the SOS study, a non-randomised case-controlled study comparing bariatric surgery to lifestyle management, demonstrated significant reductions in the cumulative incidence of macrovascular and microvascular events in the bariatric surgery group compared to a control group when followed up for up to 20 years (5). Nevertheless, this example shows that even if bariatric surgery is capable of improving HbA1c in diabetics to ‘normal’, patients remain at risk of developing microvascular complications post-operatively, and increased GV may well be a key driver for this process.

### Differences in glucose, insulin and gut hormones, between RYGB patients who have biochemical hypoglycaemia and those that do not, during a MMT

The CGM data demonstrated that patients with PPH spent 3.7% of their time in a hypoglycaemic range. Whilst these episodes occurred in the postprandial phase, they did not occur after every meal suggesting that the occurrence of hypoglycaemia is a variable phenomenon. One factor that has been postulated to precipitate hypoglycaemia is a high glycaemic index (GI) meal resulting in an increased glucose excursion followed by an exaggerated insulin response precipitating hypoglycaemia; the ‘over-swing phenomenon’. Indeed, Kellogg *et al.* has shown an improvement in PPH with a low GI diet (30). However, in this study, no significant difference in the peak glucose concentrations was demonstrated between RYGB patients that had hypoglycaemia and those that did not. The patients that subsequently had hypoglycaemia did have a significantly higher insulin peak suggesting an increased insulin response to glucose concentrations, i.e. an increased insulinogenic index.

What could be driving this increased insulin secretion? An increased GLP-1 post-prandial response has been implicated (16). In this study, we also observed an acute elevation in peak total GLP-1 concentrations within 30 min, significantly higher in those that had hypoglycaemia in response to MMT vs those that did not have hypoglycaemia. Consistent with this observation, a subcutaneous bolus of GLP-1 can induce a reactive hypoglycaemia in response to a bolus of IV glucose in people with normal glucose tolerance, i.e. an acute elevation in GLP-1 levels is able to induce an excessive insulinotropic response to carbohydrates and consequent hypoglycaemia (31). Furthermore, antagonism of the GLP-1 receptor with exendin 9–39 has been shown to rescue PPH in RYGB patients (19, 32).

On the other hand, Abrahamsson *et al.* have described cases of successful treatment of PPH with liraglutide (20), and we have observed that this is indeed effective in ameliorating PPH in one of our patients (Supplementary Fig. 1). It is counterintuitive that a GLP-1 analogue could improve PPH (33). We believe that liraglutide is effective as it is present at fairly constant levels over extended periods of time, acting to slow down small bowel transit, leading to the reduced glycaemic impact of a meal entering the jejunum via the bypass and minimizing the potential to cause hypoglycaemia (34). GLP-1’s known action of slowing gastric emptying is unlikely to contribute to its beneficial effect in PPH (34, 35, 36) as RYGB patients have a small gastric pouch, which rapidly empties, and as any acute effect on gastric motility is subject to tachyphylaxis (36, 37). Therefore, it seems that the kinetics of GLP-1 are crucial in influencing the insulinotropic response in PPH, where acute spikes can induce hypoglycaemia, but more constant levels are associated with an amelioration.

Turning our attention to the other proglucagon derivatives, we found that there was no difference in OXM secretion in response to the MMT between the MMT Hypo and MMT Non-Hypo groups. There was an early increase in glucagon secretion in response to the MMT in the PPH patients studied, and the AUC glucagon in the MMT Hypo was greater than that in the MMT Non-Hypo group. This peak of glucagon occurs prior to any hypoglycaemia, suggesting a role in the pathology of PPH as opposed to a counter-regulatory action in response to hypoglycaemia. As GLP-1 and glucagon in combination are highly insulinotropic, the coincident, early elevations in both GLP-1 and glucagon are the likely triggers of the excess insulin secretion observed in the MMT Hypo group (38).

The mechanism for elevated postprandial glucagon following RYGB surgery is not entirely known. Whilst the source of glucagon was previously ascribed to the pancreas, it is difficult to see how the early secretion of glucagon (prior to hypoglycaemia) we have observed, in parallel with GLP-1, might originate from the pancreas. More recent data have demonstrated that there is extra-pancreatic glucagon secretion in patients post pancreatectomy, who have a quite similar Roux-en-Y type bypass of the stomach to the jejeunum (39, 40). Speculatively, there may be an aberrant processing of proglucagon in L cells, leading to the early co-secretion of GLP-1 and glucagon in response to the meal stimulus prior to any hypoglycaemia.

In conclusion, these studies have demonstrated some significant findings relating to the rapid glucose fluctuations that occur in PPH and the underlying hormonal changes that drive it. There is an increased glycaemic variability observed in PPH with patients being hypoglycaemic for a significant proportion of the day. This study has highlighted an association between elevated GLP-1, glucagon and the hyperinsulinaemic hypoglycaemia observed in this condition. In future work, it will be necessary to test the effect of combined GLP-1 and glucagon antagonism in PPH patients to understand the relative contributions of these hormones in the pathophysiology of PPH.

## Supplementary Material

Supporting Figure 1

Supporting Table 1

Supporting Table 2

## Declaration of interest

All authors declare that there is no conflict of interest that could be perceived as prejudicing the impartiality of the research reported.

## Funding

This article presents independent research funded by and supported by the UK National Institute for Health Research (NIHR) Clinical Research Facility and Biomedical Research Centre at Imperial College Healthcare National Health Service (NHS) Trust. The views expressed are those of the authors and not necessarily those of the NHS, the NIHR or the Department of Health. The Section of Endocrinology and Investigative Medicine is funded by grants from the MRC, BBSRC, NIHR, an Integrative Mammalian Biology (IMB) Capacity Building Award, an FP7-HEALTH-2009-241592 EuroCHIP grant and is supported by the NIHR Biomedical Research Centre Funding Scheme.
