# Supplementary material for: Roles of increased glycaemic variability, GLP-1 and glucagon in hypoglycaemia after Roux-en-Y gastric bypass
Source: Eur J Endocrinol. 2017 Aug 30;177(6):455–64. doi: 10.1530/EJE-17-0446 (PMC5642268; doi:10.1530/EJE-17-0446)

Supplementary Figure 1 Comparison of Continuous Glucose Monitoring Traces in patient with PPH. A) Prior to Liraglutide treatment. B) During Liraglutide treatment.

A)

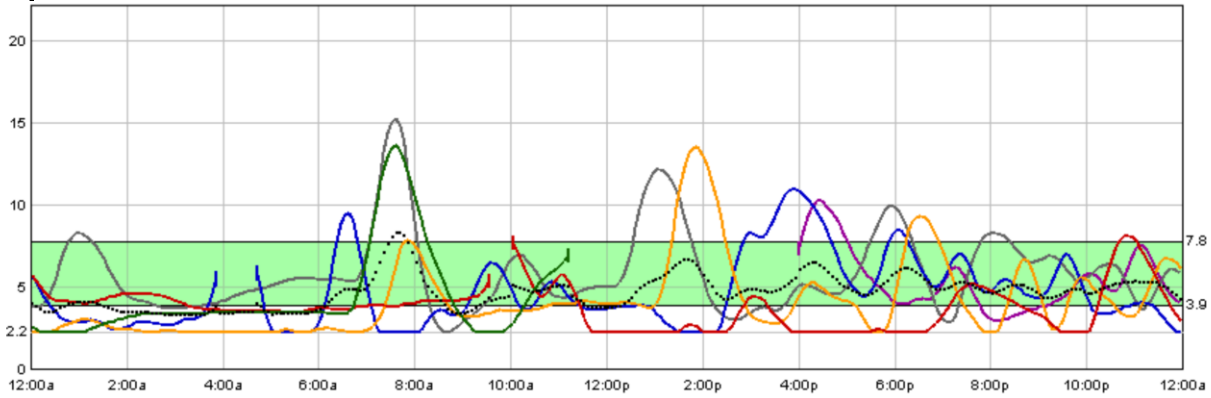

B)

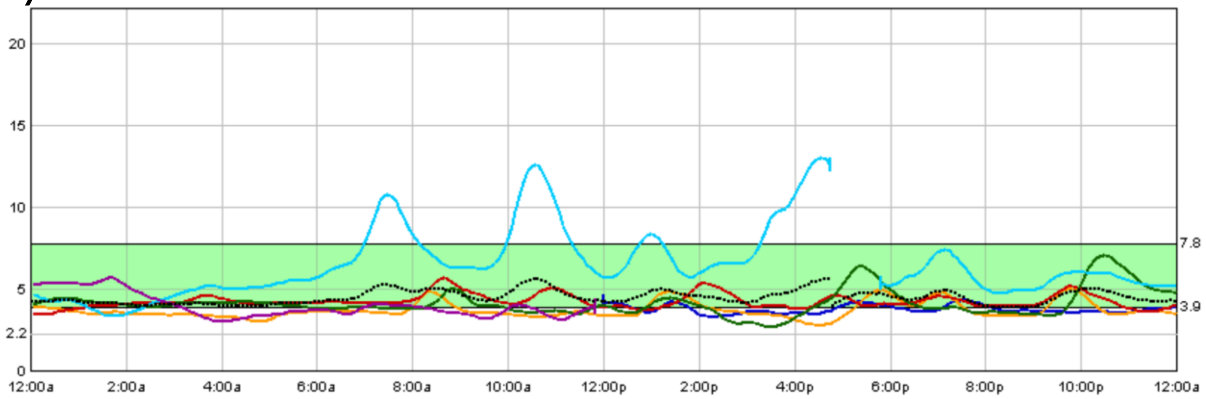

Supplement: Supporting Figure 1 [file eje-177-455-s001.pdf]
