# Supplementary material for: Roles of increased glycaemic variability, GLP-1 and glucagon in hypoglycaemia after Roux-en-Y gastric bypass
Source: Eur J Endocrinol. 2017 Aug 30;177(6):455–64. doi: 10.1530/EJE-17-0446 (PMC5642268; doi:10.1530/EJE-17-0446)
Supplement: Supporting Table 1 [file eje-177-455-t001.pdf]

Supplementary Table 1 Comparison of measures of glucose homeostasis following a MMT in symptomatic PPH group, a non-surgical obese group and an asymptomatic RYGB group.

Data expressed as mean ± SEM. Statistically significant differences vs Obese-No RYGB are shown as * = $P < 0.05$.

Outcome measures	Symptomatic PPH (N=18)	Asymptomatic RYGB (N=10)	Obese-No RYGB (N=9)
**Mean fasting glucose (mmol·l^{-1})**	4.7±0.1	5.1±0.2	5.1±0.2
**Mean peak glucose (mmol·l^{-1})**	9.3 ± 0.4	9.5±0.9	7.1 ± 0.3
**Mean nadir glucose (mmol·l^{-1})**	3.4±0.2	4.4±0.3	4.1±0.1
**Mean glucose$_{30}$ (mmol·l^{-1})**	9.0±0.4*	9.4±0.8*	6.0±0.4
**Mean time to peak glucose (min)**	35.0 ± 2.7*	36.0±19*	80.0±13.2
**Mean time to nadir glucose (min)**	140.0±11.6	132.0±21.0	220±14.1
**Mean AUC glucose (mmol·l^{-1}·min)**	1257.9±37.1	1387±57.0	1333.3±57.5
**Fasting insulin (mU·l^{-1})**	7.2±0.9	8.6±1.2	9.6±1.0
**Mean peak insulin (mU·l^{-1})**	159.0±21.3	132.4±29.6*	61.1±9.1
**Mean insulin$_{30}$ (mU·l^{-1})**	132.0±24.8*	119.4±24.9*	39.9±7.4
**Mean time to peak insulin (min)**	35.0±2.7*	36.0±4.0	73.3±12.4
**Mean AUC insulin (mU·l^{-1}·min)**	8233.4±1004.0	7706.0±2141.0	6729.2±988.9
