# Supplementary material for: Roles of increased glycaemic variability, GLP-1 and glucagon in hypoglycaemia after Roux-en-Y gastric bypass
Source: Eur J Endocrinol. 2017 Aug 30;177(6):455–64. doi: 10.1530/EJE-17-0446 (PMC5642268; doi:10.1530/EJE-17-0446)
Supplement: Supporting Table 2 [file eje-177-455-t002.pdf]

Supplementary Table 2 Comparison of markers of glucose tolerance following a mixed meal between patients who did and did not experience hypoglycaemia during the mixed meal. Data expressed as mean ± SEM. Statistically significant differences are shown as * = P < 0.05.

Outcome measures	MMT Hypo (N=7)	MMT Non Hypo (N=11)
**Mean peak glucose (mmol·l^{-1})**	9.5±0.7	9.2±0.4
**Mean nadir glucose (mmol·l^{-1})**	2.4±0.2	3.9±0.1*
**Mean time to peak glucose (min)**	30.00±0.00	38.2±4.2
**Mean time to nadir glucose (min)**	98.6±5.5	158.2±16.7*
**Glucose$_{30}$ (mmol·l^{-1})**	9.5±0.7	8.6±0.6
**Mean peak insulin (mU·l^{-1})**	232.7±25.0	112.0±21.3
**Mean time to Peak insulin (min)**	30.00±0.00	38.2±4.2
**Insulin$_{30}$ (mU·l^{-1})**	232.7±25.0	102.2±22.7*
**Mean AUC glucose (mmol·l^{-1}·min)**	1146.7±26.3	1295.4±50.0
**Mean AUC insulin (mU·l^{-1}·min)**	10327.0±763.9	7137.7±1170.7
**Mean AUC insulin:glucose (mU·mmol^{-1})**	9.1±0.8	5.5±0.8*
**Mean Insulinogenic index (mU·mmol^{-1})**	55.5±14.0	25.2±5.5*
